# “How we do it”: A qualitative study of strategies for adopting an exercise routine while living with type 1 diabetes

**DOI:** 10.3389/fendo.2022.1063859

**Published:** 2023-01-05

**Authors:** Cristine Vlcek, Dana Greenberg, Jane E. Yardley, Nika Klaprat, Andrea MacIntosh, Marley Greenberg, Joel Brandt, Natasha Gregoire, Sylvie Dostie, Denis Boutin, Conrad Pow, Mandy Archibald, Jonathan McGavock

**Affiliations:** ^1^ Faculty of Kinesiology and Recreation Management, University of Manitoba, Winnipeg, MB, Canada; ^2^ Diabetes Action Canada, Toronto, ON, Canada; ^3^ Augustana Faculty, University of Alberta, Camrose, AB, Canada; ^4^ Faculty of Kinesiology, Sport, and Recreation, University of Alberta, Edmonton, AB, Canada; ^5^ Alberta Diabetes Institute, Edmonton, AB, Canada; ^6^ Women and Children’s Health Research Institute, Edmonton, AB, Canada; ^7^ Department of Pediatrics and Child Health, Rady Faculty of Health Sciences, University of Manitoba, Winnipeg, MB, Canada; ^8^ Children’s Hospital Research Institute of Manitoba, Winnipeg, MB, Canada; ^9^ Diabetes Research Envisioned and Accomplished in Manitoba (DREAM) Research Theme, Winnipeg, MB, Canada; ^10^ College of Nursing, University of Manitoba, Winnipeg, MB, Canada

**Keywords:** type 1 diabetes, exercise, qualitative study, strategies and solutions, patient experiences

## Abstract

**Introduction:**

For people living with type 1 diabetes (T1D) the challenge of increasing daily physical activity (PA) is compounded by the increased risks of hypoglycemia and glucose variability. Little information exists on the lived experience of overcoming these barriers and adopting and maintaining an active lifestyle while living with T1D.

**Research Design and Methods:**

We conducted a patient-led qualitative study consisting of semi-structured interviews or focus groups with 22 individuals at least 16 years old living with T1D. We used existing patient co-researcher networks and snowball sampling to obtain a sample of individuals who reported being regularly physically active and had been diagnosed with T1D for at least one year. We used an interpretive description analysis to generate themes and strategies associated with maintaining an active lifestyle while living with T1D. We involved patient co-researchers in study design, data collection, and interpretation.

**Results:**

14 self-identified women and 8 self-identified men (ages 19-62, median age 32 years) completed the study, led by either a researcher, or a patient co-researcher and research assistant regarding their strategies for maintaining an active lifestyle. We identified five themes that facilitate regular sustained PA: (1) Structure and organization are important to adopt safe PA in daily life “I can’t do spontaneous exercise. I actually need a couple hours of warning minimum”; (2) Trial and error to learn how their body responds to PA and food “Once you put the time and effort into learning, you will have greater success”; (3) Psychosocial aspects of PA “…because it’s not just your body, it’s your soul, it’s your mind that exercise is for”; (4) Diabetes technology and (5) Education and peer support. Strategies to overcome barriers included (1) Technology; (2) Integrating psychosocial facilitators; (3) Insulin and carbohydrate adjustments; and (4) Planning for exercise.

**Conclusions:**

Living an active lifestyle with T1D is facilitated by dedicated structure and organization of routines, accepting the need for trial and error to understand the personalized glycemic responses to PA and careful use of food to prevent hypoglycemia. These themes could inform clinical practice guidelines or future trials that include PA interventions.

## 1 Introduction

Regular physical activity (PA) is a cornerstone to living well with type 1 diabetes (T1D) (1, 2). Individuals living with T1D who engage in regular PA experience a lower risk of complications (3), have better cardiometabolic profiles, improved quality of life and increased life expectancy (2, 4, 5). Despite these benefits, and evidence that both youth (6) and adults (7) see improvements in time spent in range (glucose levels of 3.9-10mmol/l) on active days, few people living with T1D meet current recommended levels of PA for achieving optimal health benefits (8–10). Individuals living with T1D experience the same barriers to increasing PA as the general population, for example, not being able to make time for exercise. These barriers are coupled with an increased risk and fear of hypoglycemia (11, 12) and a lack of support from healthcare providers for how to increase safely daily PA (13). Despite widespread awareness of these barriers to PA (11, 14), little information exists describing the strategies used to successfully overcome them.

Insight into the lived experience of individuals who meet the targets for recommended PA and currently maintain an active lifestyle while living with T1D could inform future behavioral change interventions or clinical practice guidelines. Qualitative methods are often used to explore contextual factors or the lived experiences of individuals that cannot be captured using traditional quantitative methods (15). Engaging individuals with extensive lived experience is a person-centered approach to intervention design can lead to more tailored, personalized approaches to behavior change (16–18). To date, there are no systematic examinations into the behaviors and strategies used by physically active people with T1D that help them achieve and sustain a lifestyle that includes regular PA. This information could help guide innovations to support inactive individuals living with T1D in adopting and sustaining a lifestyle that includes regular PA.

We adopted an integrated knowledge translation design (19, 20), whereby patient partners were involved in all aspects of the study (21). The objectives of this study were two-fold; 1) to understand challenges and successes of adopting and maintaining regular PA for people living with T1D and 2) to identify strategies for how PA can be incorporated into the lives of people living with T1D.

## 2 Materials and methods

### 2.1 Participant recruitment

A letter of invitation to participate was sent to the patient co-researchers within the Diabetes Action Canada Strategic Patient Oriented Research (SPOR) Network. Eligible participants had to be at least 16 years old, living with T1D, and self-reported being physically active for at least one year prior to enrollment. Participation was also open to caregivers of youth living with T1D. Snowball/chain sampling was also initiated as patient-co researchers reached out to physically active individuals in their T1D network and encouraged them to participate. Interested participants were screened by a research assistant to ensure they met the eligibility criteria. All participants provided informed consent. The study was carried out according to the Declaration of Helsinki (H2020:379).

### 2.2 Data collection

Participants were provided with the option of either an individual interview or a focus group interview, as patient co-researchers felt it was important to give participants the option to choose the format with which they were most comfortable. Focus groups were formed using information provided by the participant about their PA routine as strategies may be different based on frequency and intensity of the activity (1) (recreational vs competitive athlete). For example, participants who mostly walked for PA were grouped together and the participants who identified as runners were in a separate group. This separation of participants was performed at the suggestion of patient co-researchers to reduce the anxiety or fear of judgment based on PA levels within focus groups, and to facilitate comparisons related to glucose management between individuals. Focus groups consisted of two or three individuals of mixed self-identified gender.

The semi-structured interview guide was co-developed with patient co-researchers, consisted of nine open-ended questions (**Table 1**), and was designed to ask participants to reflect and recall their exercise experiences and strategies prior to the public health lock-downs associated with preventing the spread of COVID- 19. The interview guide was pilot tested by a pair of co-investigators, one research assistant and one patient co-researcher as recommended by Markula and Silk (23) to tease out confusing language, and provide feedback for any necessary changes or updates (23). After testing, the questions remained the same, but prompts were added to help further guide the discussion.

**Table 1:** Interview guide.

1. When I say the word “exercise” to you, what are some of the words or emotions that come to mind?
2. Can you describe how exercise has played a role in your life before and after your T1D diagnosis?
3. How does the management of your blood glucose levels fit within the context of exercise?
4. Can you describe some of the challenges and/or barriers you face when being physically active?
5. Can you describe some of the factors that helped you overcome those challenges?
6. Is there advice or data you wished you would’ve known or had access to regarding your own journey with T1D and physical activity?
7. Exercise programs have many “attributes” that we summarize using the acronym FITT – Frequency, Intensity, Timing, Type – such as Cardio and weights. Reflecting on your own experience and journey, can you tell me which of these attributes have worked best for you and why?
8. Reflecting on your experiences has there been a reason why the previously mentioned attributes Frequency, Intensity, Timing, Type, have not worked or will not for you?
9. Reflecting back on our conversation about barriers, FITT, and your journey with T1D and physical exercise, how do you stay motivated to remain active and engage in exercise regularly?

French language interviews were conducted by one of the researchers on the team, while English language interviews were co-conducted by a master’s level graduate student and a patient co-researcher. All interviewers disclosed whether they personally lived with T1D to each participant prior to the start of the interview. All interviewers self-identify as women and Canadian, situated in three different provinces: Ontario, Manitoba, and Alberta. All interviews were conducted *via* Zoom. Zoom was selected for its convenience and flexibility (24) as it allowed for participants with different geographical locations and time zones to be grouped together, and provided an atmosphere of inclusivity and comfort.

### 2.3 Analysis

All interviews were audio-recorded using separate, password protected devices and transcribed professionally verbatim. French transcriptions were translated into English. Participants agreed to be quoted if a pseudonym was used, and thus all participants’ names have been replaced with pseudonyms to protect identity. Data were uploaded and managed using NVivo 12. Thematic analysis was conducted using (22) five step interpretive process (23): 1) The interview was read through by members of the research team; 2) interviewers determined ‘natural meaning units’ in order to capture specific aspects of the whole; 3) dominant themes were identified and reflected back to participants; 4) the meaning of units was linked to the purpose of the study (identifying strategies to live an active lifestyle as a person living with T1D); 5) and finally, a descriptive statement based on themes was established.

### 2.4 Methodological rigor

Trustworthiness, which is the methodological equivalent of rigor (25), was established using different methods (26). Transferability of the data was important to the team (27, 28). As such, the research team invited a patient partner with extensive lived experience to help conduct the interviews in English. The inclusion of a patient co-researcher as an interviewer provided important contextual information, facilitated the interpretation and elicited more rich conversation regarding the lived experience of participating in PA. The research assistant and patient co-researcher engaged in regular discussions with members of the research team to reflect on and refine research findings. Purposeful sampling was used, ensuring participants were highly motivated individuals who could provide in-depth responses. The sample included individuals across a range of active lifestyles (e.g., daily walking to frequent high-intensity training). To ensure credibility, data analysis was conducted concurrently during data collection to explore emerging themes and to identify data saturation (the point at which the same themes were consistently emerging with new participants), which was reached after interviewing 22 participants. Data were co-analyzed with a patient partner to develop a “comprehensive, complete, and saturated” understanding of the phenomenon (25). Data were compiled into an executive summary and presented to the participants and the research teams’ wider group of patient partners for feedback as a method of debriefing.

## 3 Results

**Figure 1:**
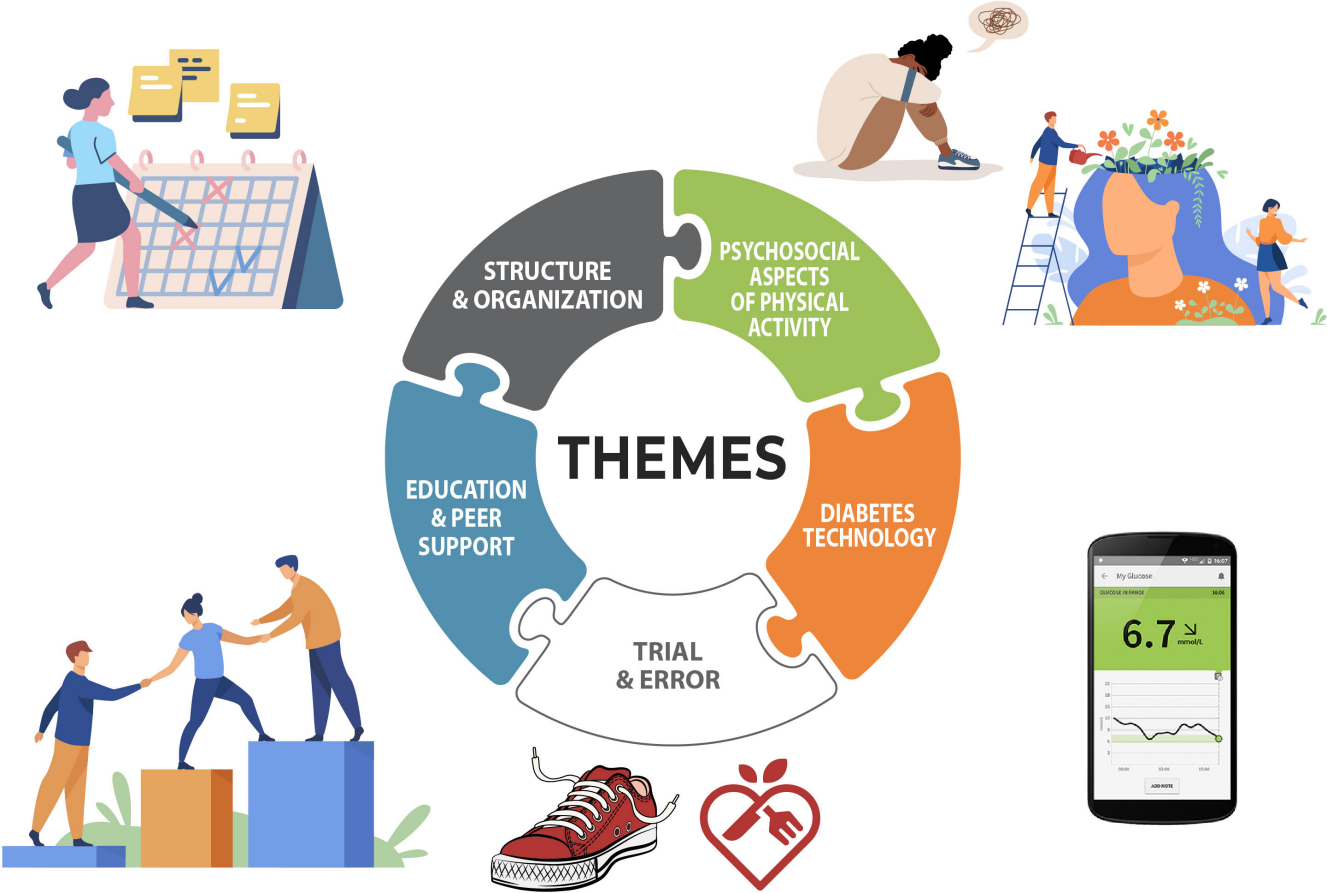
Five major themes were identified: 1) Structure & organization; 2) Psychosocial aspects of physical activity with subthemes exercise is about more than just physical health (section 3.3.1) and Emotional responses to planning for exercise (section 3.3.2); 3) Diabetes technology; 4) Trial and error related to Exercise-related trial and error (section 3.2.1 and Food as medicine (section 3.2.2); 5) Education & peer support.

**Figure 2:**
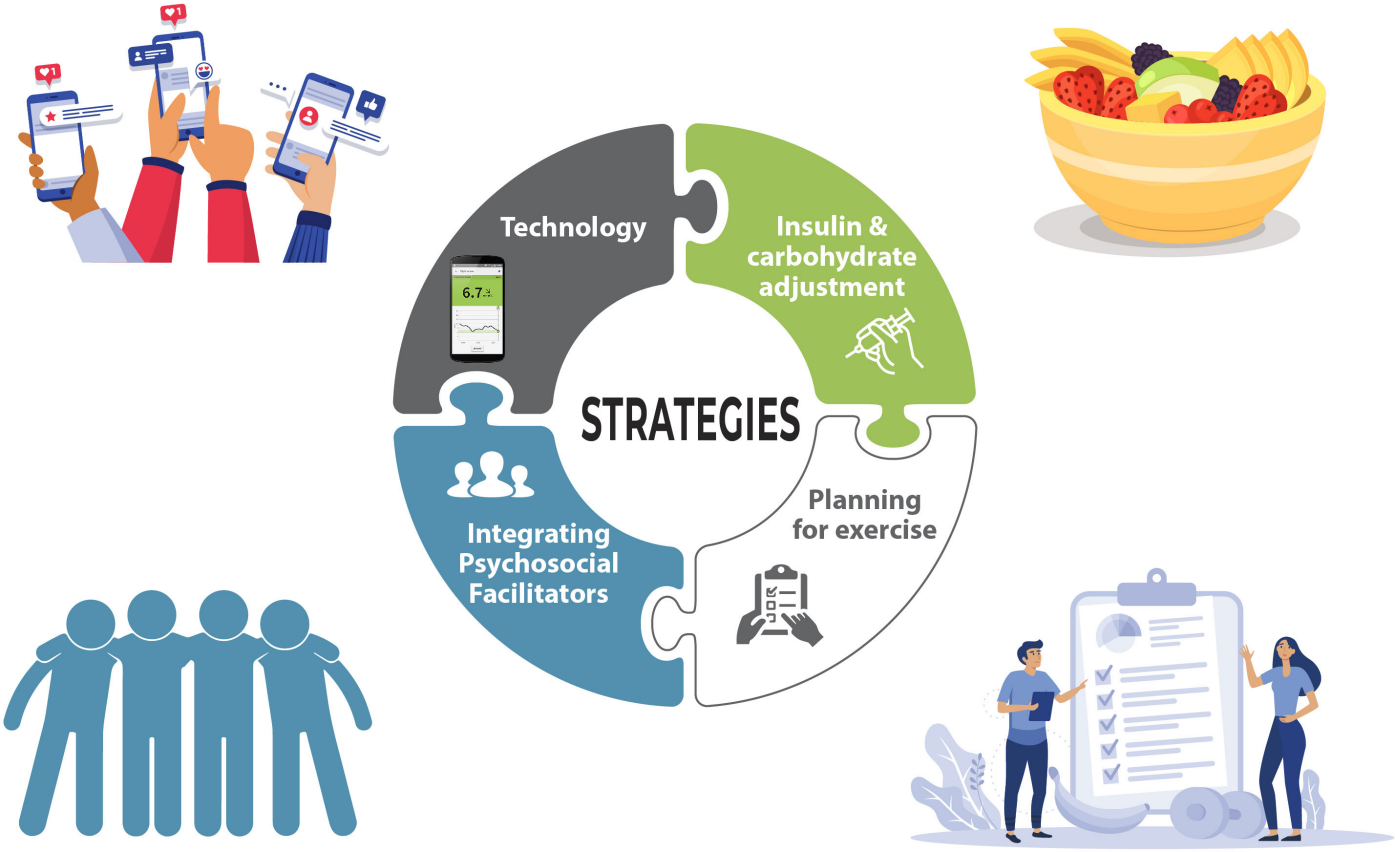
Strategies used to overcome barriers associated with exercise: 1) Technology; 2) Insulin and carbohydrate adjustment; 3) Planning for exercise; and 4) Integrating psychosocial facilitators.

Between February and July 2021, 22 people with T1D (14 self-identified women and 8 self-identified men) between the ages of 19-62 years (median age 35 years) and living in Canada (with one interviewee living in western Europe) were interviewed using Zoom videoconferencing software. Individual interviews were conducted with 11 people (n=10 in English and n=1 in French), and five focus group interviews (n=3 in English and n=2 in French). Although the study was open to caregivers of youth living with T1D, no caregivers without diabetes inquired about the study. Participants’ median age at diagnosis was 12 years, 15 reported using an insulin pump, and 19 reported having a continuous glucose monitor (CGM). All except one participant were the first in their families to be diagnosed with T1D. Participants’ activity type varied and included running, walking, yoga, resistance exercise, and a variety of sports. Based on the objectives of the study, the results are organized into two parts: 1) The themes associated with the challenges and successes of adopting and maintaining regular PA for people living with T1D (**Figures 1**, **2**) The strategies for how PA can be incorporated into the lives of people living with T1D (**Figure 2**).

### 3.1 Theme 1: Structure and organization are important to adopt safe pa in daily life

Participants emphasized the importance of organization and planning to incorporate PA safely into their daily lives. Organization was particularly important to facilitate exercising while keeping blood glucose within their preferred target range and to avoid post-exercise hypoglycemia.

“*Let’s say you and the family want to go for a walk and you’re not fully prepared for this. That can be seriously challenging. Just going for an unplanned walk, I find far more challenging than running a half marathon, if I know I’m going to do it the next day.” – Miles, male, 35*

Several participants discussed the time and planning needed to ensure that their blood glucose was within a range that was acceptable to them, to avoid a low during exercise, and to have sufficient supplies available to manage variation in the way they responded to exercise.

“*I can’t do spontaneous exercise. For me, I actually need a couple hours of warning minimum, to be able to do anything and not have it be stressful, or really messy for my blood sugars.” - Allison, female, 23*

#### 3.1.1 Other health concerns are a barrier in how to organize physical activity

Some participants noted that when choosing exercise, they are mindful of other health challenges they experienced and the difficulties/frustrations when balancing PA while managing T1D.

“*My first condition was [a chronic health condition]. So, it gives me muscle stiffness and spasms and I have to keep myself active so that my hands don’t get stiff. And then I have to deal with the stress that is brought on in terms of [chronic health condition], the difficulties.” – Olivia, female, 61*

Other participants noted how the aging process meant modifying the type of physical activity they do.

“*As I aged with diabetes, cardio was an issue and I have other chronic conditions aside from type 1 diabetes … I’m not a runner, I’m a walker” – Serena, female, 62*

One female participant simply reflected on how PA exists within a broader context influenced by other health concerns.

“*It’s not diabetes-related, but it ultimately had an impact on how I tried to manage my diabetes. My injuries have definitely been a barrier.” – Maureen, female, 20*

### 3.2 Theme 2: Trial and error

#### 3.2.1 Exercise-related trial and error

Most participants revealed that there is no ‘one size fits all’ solution for adopting and maintaining an active lifestyle.

“*We can share our knowledge, but it’s a matter of trial and error for everyone.” – Manon, female, 57*

Typical guidelines for managing the acute blood glucose response to exercise/PA did not always work for participants with T1D. Participants often expressed that even when they do find ‘a solution’, ‘a recipe’ or a routine, it may not always work for a prolonged period of time.

“*Once you put the time and effort into learning, you will have greater success in how to exercise. Even though there’s all these researchers giving you guidelines, I think as an individual, you have to do the trial and error and figure it out.” – Heather, female, 55*

“*We’re dealing more with recipes at this point, it’s more that you want to know a little bit about how we approach the challenges, but this is quite personal.” – Victor, male, 58*

Instead, participants identified that repeated sessions of the same activity while closely monitoring changes in glucose during and after activity, was essential to understanding the best approach to engage in activity and reducing the risk of glucose excursions, particularly hypoglycemia.

“*To do one at a time, not 20 takes, because you’ll never figure it out. You can, but you got to take the time and effort for each one.” – Heather, female, 55*

Participants identified several factors for the variability in their metabolic response to activity including biological (weight, puberty, injuries), social (such as being a parent with children who have varying schedules themselves or changes in work hours), and structural (reliance upon public transportation, and/or moving to a new area). As these factors are constantly changing, individuals living with T1D benefit from careful trial and error to determine the best approach to minimize glucose excursions with PA.

#### 3.2.2 Food as medicine

While guidelines are available for “healthy eating” for individuals with T1D (29), participants felt that food was medicine for them and trial and error was important to “titrate” appropriate food types, carbohydrate concentrations/amounts and timing of intake to manage their glucose response to different food types before, during and following PA.

Participants felt that food is deeply interconnected with PA when living with T1D, to a much greater degree than individuals without T1D.

“*I think that from the moment I was diagnosed, I guess I just became really type A about what I ate and how it made me feel. Because it greatly influenced what I was able to do in a day, whether it was like even just studying or exercising. Yes, I think I’m super aware of my body now. Especially how it feels, not its physical appearance, but how I feel inside my body.” – Riley, female, 22*

Participants with T1D reported managing unpredictable, at times extreme, changes in blood glucose with PA that require precise amounts of carbohydrates (and at times insulin) to correct.

“*If it’s a day where I know I’m going to be sitting in a classroom, I don’t really want to eat 100 grams of carbs for breakfast, because I’m just going to be asleep throughout the day. Whereas, if it’s a day where I’m going to be hiking or stream fishing, then I can eat 100 grams of carbs and not really even use any insulin and just go the whole day.” – Jack, male, 35*

### 3.3 Theme 3: Psychosocial aspects of physical activity

#### 3.3.1 Exercise is about more than just physical health

For individuals living with T1D, PA represented more than a tool for self-management behavior and improving their physical health. To them, exercise was embedded in their socio-cultural context.

“*…because it’s not just your body, it’s your soul, it’s your mind that exercise is for. It’s not just being fit. It clears my head. I feel like I can think and accomplish more once I get on a level of doing a bunch of classes. It just frees me in so many ways beyond just a healthy physique.”–Serena, female, 62*

Physical activity was also viewed as essential to supporting positive mental health by creating spaces and times to reflect, bond and socialize with others.

“*What has changed, exercise, it’s good for my mental health. Exercise for me has become like a drug. If I don’t do it, I really don’t feel good. It is something that I need every day.” – Manon, female, 57*

“*I am a social creature; I hate doing stuff by myself. No, I love doing things together, I’m a fan of team sports. Like playing golf with the right group of guys, oh boy, that’s a great afternoon, and it’s awesome.” – Tyson, male, 51*

#### 3.3.2 Emotional responses to planning for exercise

Interviewees highlighted that being physically active while living with T1D is not an independent event, as PA or exercise is often marketed as ‘leisure’ or separate to an individual’s academic schedule or employment.

Pre- and post-exercise organization and emotional responses were identified in the preparation to participate in a planned activity, as well as how that activity negatively affected other areas of the person’s life.

“*The emotion is groaning. It’s more of a reluctance knowing this is going to take work and exhaust me and not just physically, not in the positive exercise sense, but take a toll on my body and potentially inhibit my ability to do my homework or my other things later because my blood sugars are going to be a mess.” – Eileen, female, 21*

“*When I was younger, I didn’t really understand the emotions of diabetes, I guess, with going low and going high. It was still something my parents would see the signs of. I wasn’t fully aware of it as I am now. It’s something that I’m more aware of now and I’m trying to figure out a way to walk without going low, but it’s a learning process.” – Jillian, female, 20*

One participant noted that even though the reason they engage in PA was because they know it was beneficial for them, the overall feeling of stress was dominant.

“*For me, it’s less positive. For me, it’s a source of stress though. It’s about well-being as well, I do it for well-being, but it’s also a matter of stress, it’s there quite a bit when I think about exercise.” – Bridget, female, 50*

### 3.4 Theme 4: Diabetes technology

While lifesaving and changing, participants in this study spoke about how T1D medical devices such as insulin pumps and continuous glucose monitors could act as a barrier due to the logistical challenges of certain sports, the participants’ geographical locations, and weather conditions.

“*…it’s also just playing volleyball with the pump on, it doesn’t work. I don’t want to break it, because you dive pretty much on every side of your body. I don’t want to land on it. It hurts, too. It’s a little plastic thing that digs into your hip bones, so it also hurts. I’ve tried it, but it didn’t work for me” – Robert, male, 19*

“*I’d say, for me, the biggest issue … is, with these sensors and the way the adhesive works … the tiniest little bit of sweat will make the adhesive not work anymore … I almost lost the transmitter a few times” – Aaron, male, 24*

Participants spoke about the fear of theft and the many costs of replacing devices can be added to the stress associated with daily PA. Clothing can act as a barrier, as many sports apparel are tight-fitting, and fail to take into account the need for extracorporeal devices.

“*My friends want to go into the water and I’m really reluctant to go into the water because I don’t want to take it (my pump) off. My pump’s not waterproof. I took off my pump and my phone, but I had to be able to see it because I’m super paranoid that someone’s going to take it, which is not a ridiculous paranoia.” – Eileen, female, 21*

### 3.5 Theme 5: Education and peer support

Participants stressed the importance of having knowledge about T1D to understand how to incorporate activity into daily life.

“*I probably wish I had been more informed and aware of that (exercise) and open to it at a younger age. I think it would have made a difference…”-Serena, female, 62*

“*I think just even some really basic stuff is missing on activity. Although everybody is really different, I think having some just really basic guides on like, “Hey, if you want to start running, here are the things you should probably be looking for.” – Lawrence, male, 35*

One concern that was raised by two participants was regarding the lack of information or education about the differences between type 1 and type 2 diabetes. The first participant raised it in the context of information she personally didn’t receive from her healthcare providers.

“*I got no data. Any pamphlet that I got was type 2…it was something that really bothered me at the time because it was normally like, ‘Oh, this is a good way to manage your blood sugars without resorting to insulin if you were type 2.’ I was like, ‘I’m athletic already. I didn’t get type 2’.” – Riley, female, 22*

The second participant expressed frustration about other people’s lack of knowledge about type 1 diabetes.

“*They don’t understand. I’m kind of tired of educating everybody all the time. I feel like sometimes people think it’s not that bad because they know type 2 diabetes. They see that even if the type 2 diabetic forgot his medication or this or that, it’s not that bad, he/she will be able to use another alternative solution. For us as diabetics, it’s different.” – Bridget, female, 50*

Another female participant reflected about how being able to connect with others using technology to share information has helped her to be able to advocate for her child living with T1D.

“*Yes … The online community has made a huge difference for parents and caregivers, because it’s helped people become that much more of their own advocates for their children, never mind their own…” – Heather, female, 55*

Participants also discussed the importance of having access to peers with whom they can share their knowledge.

“*I think a diet buddy or something like that for your first three months. To be honest, I don’t know how I didn’t die.” – Jack, male, 35*

In addition to these main themes, participants identified specific factors that helped them overcome barriers to PA (question number five in **Table 1**). The use of technology, and psychosocial factors - specifically support from friends, family, spouse, and others with T1D, were the most common factors identified. The support from other people also extended beyond the physical presence of someone else, to the social aspect and accountability that exercising with others brings. Strategies regarding carbohydrate consumption and insulin dosing, as well as the need to plan ahead for exercise were also specific strategies participants discussed. The strategies identified relate to the main themes.

### 3.6 Strategy 1: Technology plays an important role in facilitating exercise

Participants reflected on how management of their diabetes during exercise changed for the better after they started using a continuous glucose monitor (CGM). In some ways the CGM made exercise itself easier, and for others it made the planning process less cumbersome.

“*At the time when I first started exercising, I didn’t have a CGM and I think that that’s been really impactful, to be able to see the effects more clearly.”-Lawrence, male, 35*

“*The spontaneity is definitely; it’s become easier now that I have my CGM. As long as I have my phone within range for a recreational activity or something, I’d probably keep my phone in my pocket and just monitor myself. If I got down to even four or five even, I may correct it a little earlier than normal.” – Penelope, female, 27*

Other participants agreed that advances in technology have helped in the management of their diabetes, so has the use of social media.

“*But technology has helped me a lot, and so has my wife. It was much harder to manage my diabetes when I was 25 than it is now … There’s also the Facebook group where I saw the research, I thought it was wonderful.”– Paul, male, 43*

### 3.7 Strategy 2: Integrating psychosocial facilitators

Participants spoke about a variety of factors, some that directly facilitate exercise, and others that motivate and uplift them, making living with diabetes (and thus exercise indirectly) a little bit easier.

Participants spoke about having a support system when exercising being a strategy that helps them. One participant spoke about family members, whereas another participant liked getting to know people at his gym, and the sense of accountability that he felt to exercise.

“*I’ve always been participating in sports and team sports. The atmosphere of games, family, because my family always came to our games, there’s a huge gathering all my aunts and uncles to come.” – Maureen, female, 20*

“*Going to a gym, another benefit would be the social aspect. Social aspect, you see people, you can communicate with people, as well as whether it’s an official accountability program or not … they just expect you to show up.” – Miles, male, 35*

One participant spoke fondly about her friends, and how they are linked in to her CGM and actually help her manage her blood sugars.

“*I’m the luckiest person in the world. I have three friends that are linked to my Dexcom. We do have a very nice protocol. It took us like six months to get it right.” – Violette, female, 32*

One person felt very alone and spoke on two occasions about how being able to connect with others living with T1D is a very important strategy for them.

“*…someone who told me about a Facebook group … then it was so much of a revelation to see that I’m not all alone that there are other worlds like me, that have the same challenges that I do, then to be open to learning then not just remain stuck with myself….” – Caroline, female, 45*

“*For me it’s really, you learn more from people who have diabetes like you. Typically, one person will get coffee, the blood sugar level will go rise, for another person it may go down. At least you know you’re not alone. That’s really, for me, the main key.” – Caroline, female, 45*

The feeling of not being alone with their diabetes and learning from peers with T1D was also echoed by others. One participant even spoke about the interview for our study being a place where she learned something new through a peer.

“*That’s something that also helps, to meet people who are going through the same thing as we are, to give them tips, because for me, just waiting to hear about the pump today makes me wonder why my doctor didn’t tell me about all this.” – Bridget, female, 50*

### 3.8 Strategy 3: Carbohydrate and insulin adjustments

One participant discussed the role her friends played in making her realize she needed to make some carbohydrate modifications.

“*It wasn’t even me, it was one of my friends who was like, ‘Look, what is the problem here? Your blood sugar goes up when you do what? When you eat carbs? Could you just get rid of that?’…Then you realized that you can.”– Violette, female, 32*

Others brought up feeling the need to makes change to their insulin and carbohydrates to avoid further complications. One participant said:

“*When it started [exercise], … I felt I’ve probably done some physical damage and complications due to diabetes … Since then, I have been very intentional of switching over to a very low-carb diet. I changed my insulin regime, 99% of the time, I’m just on regular insulin, very slow acting.”– Miles, male, 35*

### 3.9 Strategy 4: Planning for exercise

Within the context of Theme #1, structure and organization, many participants described the importance of planning for exercise. Regular structure for the time of day and type of activity being performed allowed for careful titration of insulin and meal planning. Spontaneous activity was significantly more challenging due to uncertainty of the personalized glycemic response to PA.

“*My friends know that I have to plan. I have to have a certain amount of time to figure everything out…” - Allison, female, 23*

## 4 Discussion

The aim of this study was to identify strategies associated with adopting and maintaining an active lifestyle for individuals living with T1D. Using semi-structured interviews, we identified five themes: Structure and organization are important to safe PA in daily life; Trial and error; Psychosocial aspects of PA; Diabetes technology; and Education & peer support. Specific strategies used to overcome exercise-related barriers were related to technology, integrating psychosocial facilitators, insulin and carbohydrate adjustments, and planning for exercise. These themes and strategies could serve as a starting point for developing interventions or clinical targets for supporting people living with T1D to adopt and maintain a more active lifestyle.

A recent systematic scoping review of barriers to PA for individuals with T1D (11) identified commonly cited barriers, including the increased demands placed on individuals such as the extra time, planning and energy required to safely engage in PA. The theme of trial and error was also a commonly cited barrier that was unique to individuals living with T1D. A significant amount of individual trial and error is required to learn the different factors that influence how the body responds to different types of exercise and how to manage the different changes in blood glucose (30–32). In our study, Structure and Organization, and Trial and Error were identified as methods to adopt and sustain an active lifestyle. Interestingly, rather than viewing trial and error as a barrier, participants in our study viewed it as an essential component of adopting an active lifestyle. The other common thread in our study and others (30–32), was that trial and error was sometimes described as a challenge/barrier, but also a necessary process or strategy that leads to feelings of success and mastery. Despite a broad range of evidence-based strategies for preventing hypoglycemia during an acute exercise session (1, 33), participants did not discuss using them, and still felt that a lengthy trial and error process was required. Future work is needed to understand the determinants of successful and efficient trial and error processes used to facilitate safely engaging in PA for individuals living with T1D.

Another commonly cited barrier to exercise is the lack of guidance from healthcare professionals (11, 13). This often takes the form of a general absence of support, guidance or information given on how to navigate exercise. This theme was also echoed in our study in the Education and Peer Support theme where participants noted the lack of information about how to use food to exercise safely, and how to approach exercise. Related to lack of information was the frustration expressed by our participants about lack of knowledge from family/friends/the community in general about T1D, which negatively affected participant social/emotional wellbeing. Similar findings were reported in a qualitative study of adolescents living with T1D (34). Adolescents reported a lack of knowledge about T1D among school staff and peers, which can serve as a barrier to exercise in schools. This reinforces the importance of peer support and receiving knowledge from others with similar lived experience. In the absence of information for optimal strategies from health providers, the T1D community was an important source of information to facilitate and support the adoption of an active lifestyle. These data support the importance of peer support in the design of interventions aimed at increasing PA for individuals living with T1D.

Psychosocial factors are an important determinant of engaging in regular PA for people living with diabetes (35). Similar to previous studies, participants described the emotional burden associated with living an active lifestyle while trying to avoid hypoglycemia. This emotional burden is intimately linked with the fear of hypoglycemia (12) and unpredictability for the glycemic response to an exercise session. The participants in this study mainly spoke about psychosocial factors as being facilitators to PA or ways they overcame challenges they faced with exercise, specifically peer and family support. Participants also described a sense of gratitude and joy that was gained from participating in PA. Importantly, participants reflected on these benefits while not talking about the health impacts or benefits for preventing diabetes-related complications. These findings build on previous surveys of people with T1D, which revealed that a sense of self-efficacy and intention were predictors of PA behaviors (36, 37). Our findings also reinforce previous qualitative studies that identified the importance of social support and motivation as drivers of PA (31, 38). Similar previous studies in adolescents with type 2 diabetes (39) report a connection to others was an important facilitator to PA. In our study, connecting with others with T1D (i.e. peers) to share knowledge and experiences, the support of a friend or family member, or participating in team sports fostered a sense of connectedness and accountability that made it easier to incorporate PA into their lives. This information could help guide messaging for supporting behavior change for people living with T1D, by focusing on the psychosocial benefits of PA, rather than the physiological benefits.

Common strategies for preparing for exercise for people with T1D include 1) more frequent glucose checks (40), 2) using additional carbohydrates before, during, and after exercise (40, 41), and 3) decreasing insulin before and after exercise (40, 41). The participants in our study did not report checking glucose more frequently. The majority of the participants were using a CGM though and would not have reported more frequent self-monitoring blood glucose checks with a lancet and glucometer. Nobody reported more frequently checking CGM glucose levels either. However understanding personalized carbohydrate and insulin adjustments for different types of PA was viewed an important strategy to maintain an active lifestyle. Specifically, the participants in this study focused more on how they made adjustments to insulin and carbohydrates in the larger context of their lives, and did not provide many specifics that relate to the above mentioned strategies. Future studies could focus on understanding the personalized approaches that individuals adopt to learn how best to adapt carbohydrate and insulin adjustments, as few individuals use currently published guidelines (42).

The other major factor or strategy that helped our participants overcome challenges with exercise was technology. Interestingly, technology emerged in a mostly negative context (for example, the adhesive binding a CGM sensor to the skin coming loose when sweating, or an insulin pump being cumbersome to wear while playing volleyball) in the general conversation but came up exclusively in a positive manner when participants were asked specifically what helped overcome barriers. A large proportion of the discussion was related to diabetes-specific technology such as the ease of monitoring blood sugars with the use of a continuous glucose monitor (CGM), but also the use of online fora to connect with others to learn. This was in contrast to other studies (30–32, 43, 44), where technology was not at the forefront of how to navigate exercise.

Strengths of the present study include co-designing the study with people living with T1D, having one of them to help conduct the interviews, and engaging a diverse sample of participants with extensive lived experience of being physically active with T1D. Limitations include the use of snowball sampling which can introduce “selection bias” and limit the breadth of themes that emerge due to a homogenous interconnected sample of participants. This may explain why gender or sex-specific factors did not emerge from interviews and focus groups, as there are indications from survey data that gender may play a role in how individuals with T1D undertake exercise (40). Second, we were not able to recruit caregivers of someone living with T1D, although the study was open to caregivers. We were therefore unable to triangulate information provided with other factors that a caregiver may experience or observe. Third, we did not observe differences in themes or strategies between individuals on automated insulin delivery and those on multiple daily injections, however, we were likely underpowered to identify specific differences. Lastly, the study was conducted during the COVID-19 pandemic. While this situation allowed for greater diversity in the participants being studied because participants could login virtually to participate, it may have influenced the depth of the discussions as interviews were not conducted in person. Despite these limitations, the data presented here provide the first in-depth examination into the strategies that have been used by people living with T1D to adopt and maintain an active lifestyle. These could inform future exercise or PA interventions or clinical care guidelines for diabetes educators.

## 5 Conclusion

Qualitative investigation into an individual’s lived experience can identify novel targets for future interventions. This study highlighted the discrepancy between reliance on one-self, the significant amount of trial and error needed to navigate exercise, and the desire for more connectedness with others through the support of others and technology. The latter are important features to consider in the design of future exercise interventions in people living with T1D.

## Data availability statement

The raw data supporting the conclusions of this article will be made available by the authors, without undue reservation.

## Ethics statement

The studies involving human participants were reviewed and approved by the University of Manitoba Bannatyne Campus Health Research Ethics Board. The patients/participants provided their written informed consent to participate in this study.

## Author contributions

JM, JY, MA, NK, DG, MG, JB, SD, DB, CP, and NG contributed to the study concept and design. CV, DG, and JY collected the data. CV performed the data analysis. JM, CV, DG, and AM participated in data interpretation. The manuscript was drafted by CV, AM, and JM and reviewed by all authors. All authors contributed to the article and approved the submitted version.
